# Participation in the “nutrition at the Centre” project through women’s group improved exclusive breastfeeding practices, as measured by the deuterium oxide dose-to-mother technique

**DOI:** 10.1186/s13006-020-00302-y

**Published:** 2020-06-26

**Authors:** Jaures H. F. Lokonon, Waliou Amoussa Hounkpatin, Nicole Idohou-Dossou

**Affiliations:** 1 School of Nutrition, Food Sciences and Technologies, Faculty of Agronomics Sciences, University of Abomey-Calavi, Abomey-Calavi, Republic of Benin; 2 Laboratory of Nutrition, Department of Animal Biology, Faculty of Sciences and Techniques, University Cheikh Anta Diop, Dakar, Senegal

**Keywords:** Deuterium oxide, Exclusive breastfeeding, VSLA groups, N@C project, Educational intervention, Benin

## Abstract

**Background:**

Evidence of interventions that are effective in improving exclusive breastfeeding (EBF) practices is needed to help countries revise their strategies. To assess whether mothers who had participated in the Nutrition at the Centre (N@C) project effectively demonstrated better EBF practices than did those who did not participate, we documented the processes of this nutritional intervention in Benin.

**Methods:**

This study was a cross-sectional design comparing the intervention group, namely, the Village Saving and Loan Association (VSLA-N@C), to the control group. The N@C project was an educational intervention based on behavioural and social changes related to nutrition. Through VSLA groups installed in communities, mothers were connected to the project; had weekly discussions around the process, benefits and challenges linked to EBF, and advocated during Breastfeeding Week celebrations. The study participants were mothers with children aged 4–5.5 months from the VSLA-N@C group (*n* = 53) and mothers (*n* = 50) from non-intervention areas who served as controls. With the deuterium oxide dose-to-mother technique, we quantified human milk intake (HMI) and non-milk oral intake (NMOI) and compared both groups using Student’s *t*-test. A child is considered to be exclusively breastfed if the NMOI is less than 86.6 g/day. Multivariate regression logistics adjusted for VSLA membership, mothers’ body mass index, and children’s age, weight-for-age and weight-for-length, thus enabling us to measure differences in EBF rates.

**Results:**

Children of mothers from the VSLA-N@C group consumed significantly more human milk than those of mothers in the control group (900.2 ± 152.5 g/day vs 842.2 ± 188.6 g/day, *P* = 0.044). Children in the VSLA-N@C group had significantly less non-milk oral intake than did those in the control group (difference: 148.2 g/day, *P* = 0.000). Therefore, the EBF rate was significantly higher in the VSLA group (38% vs 8%, *P* < 0.0001), and mothers in VSLAs were 14 times more likely to practise EBF than were those in the control group (adjusted odds ratio [AOR] = 13.9, 95% CI 1.9–116.5, *P* = 0.015).

**Conclusion:**

The EBF rate was significantly higher in the group of mothers who participated in the VSLA-N@C project than in those who did not receive the intervention. The N@C model could be promoted as a strategy for increasing EBF practices in poor and rural contexts, where it is possible to organize mothers into VSLA groups to discuss the process, benefits and challenges of EBF.

## Background

Exclusive breastfeeding (EBF) during the first 6 months of age reduces the incidence of diarrhoea, acute lower respiratory infection and infant mortality [1]. It is expected that breastfeeding promotion strategies will have an impact on children’s nutritional status by preventing both growth deficits in children and overweight and obesity later in life [2]. Despite these advantages, many developing countries, such as Benin, still have an EBF rate of under 50%, which is one of the six major global targets of the United Nations Decade of Action on Nutrition [3]. According to Demographic and Health Survey (DHS) data, the EBF rate in Benin varied from 33% in 2011 [4] to 42% in 2017 [5]. In a sub-region of Benin, where the isotope method was used to objectively determine the prevalence of EBF, only 3.5% of mothers breastfed their babies exclusively [6]. Therefore, the identification of interventions that are able to increase EBF rates is important for the country, as a lack of EBF also presents a high prevalence of stunting (> 30%) for 10 years [5]. To reduce child stunting, CARE International implemented a 5-year integrated nutrition programme called Nutrition at the Centre (N@C) in Benin, Bangladesh, Ethiopia and Zambia from 2014 to 2017 [7]. The N@C project also documented evidence, lessons learned and good practices in each country to globally inform future nutrition programming and influence national and international strategies for improving nutritional outcomes (e.g., EBF, anaemia, stunting) [7].

In Benin, EBF promotion has been central in CARE activities within communities where beneficiaries’ women were organized into a Village Saving and Loan Association (VSLA-N@C). Through these women’s groups, they were connected to the N@C project and engaged in weekly discussions about nutrition outcomes. Particularly for EBF, the discussions were around the process, benefits and challenges linked to EBF, which were advocated during Breastfeeding Week celebrations. Therefore, the evaluation of this approach to precisely test the adoption of EBF behaviour is critical in generating evidence and reinforcing the success of the integrated approach of the N@C project.

The evaluation of education and counselling programmes is usually based on maternal report, which is easy and cost effective to obtain but has major limitations. A review of studies conducted in numerous developing countries using the isotope technique to validate mothers’ reports has shown that the discrepancy between mothers’ recall (24 h) and the deuterium oxide dose-to-mother technique (DTM) can be as high as 40% at 3–6 months of age [8–12]. There is a discrepancy between mothers’ recall and DTM because mothers tend to over report their adherence to the practices to gain the approval of the interviewer or underreport because of lapses in memory [13]. A recent publication from Mulol and Coutsoudis revealed an overreporting of EBF at three different time points (6 weeks, 3 months and 5.5 months) regardless of the cut-off value used to assess EBF by the stable isotope technique [14]. These authors suggested that the more objective gold-standard stable isotope method should be used to evaluate interventions with smaller representative samples [14]. Here, DTM was used to objectively assess whether the mothers who participated in the VSLA-N@C group had better EBF practices than those who did not participate and lived in non-interventional areas. It is expected that this knowledge will contribute to the evaluation of the impact of the N@C project on EBF practices.

## Methods

### Summary description of the N@C project

**Fig. 1:**
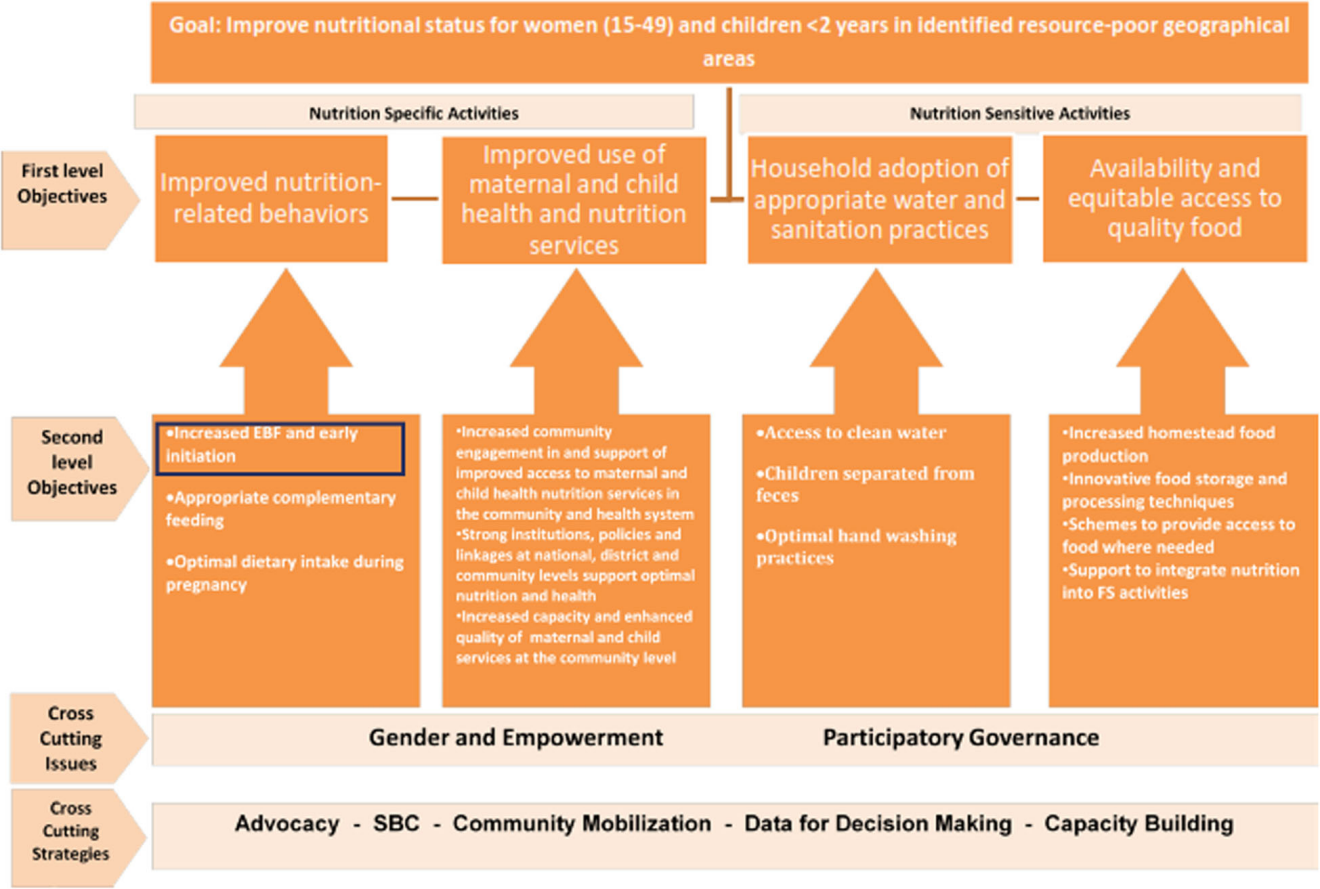
Results framework for the Nutrition at the Centre (N@C), CARE International. BSC: Behavioural and social change; FS: Food Security

The N@C project included the promotion of infant and young child feeding practices, the improved use of maternal and child health and nutrition services, access to quality food, the household adoption of appropriate water and sanitation practices and women’s economic empowerment and gender equity in poor settings (Fig. 1). The N@C project was implemented in two communities in southern Benin (Dangbo and Bonou) and in collaboration with different stakeholders, such as the National Council of Food and Nutrition, the Ministry of Agriculture, the Ministry of Health, the Ministry of Social Affairs and Family, UN agencies, and international and national NGOs.

The messages of communication for behaviour and social change were conveyed through the VSLA (women’s group) by field workers and catalysts from communities. Several methods and approaches are combined to promote EBF practices within the N@C project. We have i) social communication in the women’s group (VSLA) to promote periodic awareness among members, ii) home visits in the households of women’s group members by project facilitators and catalysts for counselling according to need and to inform and sensitize non-members of the VSLA group; iii) sketches, games and songs promoting awareness on a sub-theme previously defined by the management team; iv) community videos, including film screenings on the advantages of EBF and proper positioning and support of lactating mothers in all intervention villages; v) dissemination of messages to promote good EBF practices by local radio stations; and vi) public events, such as community durbars (*Atchapkodji*) and annual World Breastfeeding Week celebrations, as opportunities for the promotion of breastfeeding on a large scale through various activities with the involvement of communities and local authorities. More details on the N@C project can be found in a previous publication [7].

After participating in a session in the VSLA group, the mothers were invited to share the information they received with other mothers. This study focused only on mothers who were members of the VSLA groups.

### Design and study area

This was a cross-sectional study conducted in the intervention group of the N@C project (VSLA-N@C) and the control group.

Intervention groups were mothers’ groups implemented in all sixteen (16) villages of Dangbo commune, in which the N@C had implemented its activities. Before this study, a pilot that showed the critical points and challenges of using the deuterium oxide dose-to-mother technique and the dilution technique among Beninese children was conducted in the Bonou community, and the results were published [15].

The control group included mothers who were selected from another community located at least 100 km from Dangbo in southern Benin, where no intervention had taken place or was likely to take place before the end of our study. The distance was to limit the possibility of diffusion of N@C messages and approaches from implementation zones. The district of Tori-Bossito was selected based on the fact that the population has a similar food situation, agroecology and nutritional status as the control group [16]. The only EBF message exposure and promotion to mothers in the control group were the messages delivered by government agents.

#### Recruitment and selection of participants

A list of mothers with healthy children aged 4 to 5.5 months in the villages of two districts of Tori-Bossito (Tori Cada and Azohoue Cada) and two districts of Dangbo (Zoungue and Hozin) was extracted from vaccination cards in each of the health centres of these localities and complemented by available birth records at the local administration. All mothers of the intervention group were members of the VSLA-N@C project. The mothers and their children were selected according to the inclusion and exclusion criteria.

#### Inclusion and exclusion

All lactating mothers with children aged four to 5.5 months who agreed to participate in the study were included.

The exclusion criteria were as follows: participants who were sick or refused to participate in the study; mothers with multiple births and their babies; children suffering from severe malnutrition after the first control; children suffering from diseases requiring hospitalization; mothers under 16 years old; and mothers who have lived in the community for less than 6 months.

### Sample size

The size was calculated by using a published formula [17] based on the objective of comparing the rate of EBF practices between the VSLA–N@C and control groups. The rate of EBF practices among mothers with children aged 4–5.5 months was 25% [18]. With the intensive activity of the N@C project, we aimed to detect at least 25% of the difference between the two groups. Thus, for 80% power and a significance level of 5%, we needed a minimum of 45 mothers per group. Considering a 20% drop-out rate, we retained 54 mothers per group. This size was consistent with a 100 g/day detectable difference in milk intake between exclusively breastfed and non-exclusively breastfed children, for a standard deviation of 130 g/day of milk intake [19]. Considering all inclusion criteria, the final sample size for the study was 103 mother and infant pairs, with 53 in the intervention group and 50 in the control group.

### Data collected

#### Determination of exclusive breastfeeding practices using DTM

The standard operating procedure of using DTM has been largely explained by the International Atomic Energy Agency [19] and is detailed in much of the existing literature [8, 9, 11, 12, 20–23]. Briefly, after pre-dose saliva collection, a 30 g dose of deuterium oxide was administered, and post-dose saliva samples were collected in cryogenic tubes from the mother (~ 4 ml) and child (~ 3 ml) pair on the 1st, 2nd, 3rd, 4th, 13th and 14th days after dose administration to the mother. Deuterium enrichment of saliva was measured using a Fourier transform infrared spectrophotometer (FTIR Shimadzu Prestige 21). All saliva samples were analysed in duplicate. All enrichment used for the analysis had a coefficient of variation under 1% [19]. The field data (weight, height, age, hour of collecting saliva, dose weight) and laboratory data (enrichment measurements) were recorded in an Excel program designed to calculate human milk; the data were entered by two research assistants and verified by a third assistant to limit biased entry. The Solver function of Excel® was then used to fit the deuterium enrichment of the saliva samples from the mother and the infant over the 14-day period to model curves. The outputs include breast milk intake (labelled water) and non-milk oral intake (NMOI, i.e., water from sources other than breast milk, which is unlabelled). We considered a child exclusively breastfed if the non-milk oral intake was less than 86.6 g/day [24]. This high cut off point (86.6 g/day) is needed due to measurement error and others issues linked to the demanding and challenging saliva collection for the mothers and their babies (seventeen saliva samples in total), relative to the use of the DTM. Liu et al. [24] explained that this cut off would be the sum of theoretical and experimental error introduced in the field work that propagates into the analysis.

#### Anthropometry

Children’s weight was measured by a SECA 354 scale with an accuracy of 0.01 kg, and mothers’ weight was assessed by a SECA 869 scale with an accuracy of 0.1 kg. The length of the children and height of the mothers were measured using a ShorrBoard. The mean anthropometric indices of the children, namely, length-for-age (LAZ), weight-for-length (WLZ) and weight-for-age (WAZ), were generated by WHO Anthro [25].

#### Questionnaire

Questionnaires were administered to mothers to collect information on socio-economic status, household food security [26] and participation in N@C activities (in the intervention group) or similar activities (in the control group) during the last 6 months. In addition, the standard WHO questionnaire to evaluate EBF from the 24-h recall of mothers was used [27]. All information was collected on the first day (day 0) of the study using electronic tablets. The socio-economic status of households was calculated on the basis of goods and materials that the household owns [18]. Household food insecurity was estimated by an ordinal scale variable determined with the Household Food Insecurity Access Scale [26].

### Data analysis

A chi-square test was used to compare the characteristics of household, children’s and mothers’ participation in activities and EBF practices between the two groups. For continuous variables, such as anthropometric indices, human milk intake, and non-milk oral intake, Student’s *t*-test was applied.

A regression logistic multivariate analysis was conducted to determine an odds ratio associated with the benefit of intensive promotion of EBF represented by group. Variables for adjusting the model included VSLA membership, mothers’ body mass index (BMI), and children’s age, WAZ, and WLZ. A level of 5% was considered significant, and all analyses were performed in STATA 13 software.

## Results

### General characteristics of participants

Table 1 presents the general characteristics of the participants and their households. There were no significant differences in food security or socio-economic status between the VSLA-N@C and the control groups. Approximately 96 and 98% of households were food insecure in the VSLA-N@C and control groups, respectively. The percentage of households with poor socio-economic status was 94.2 and 86% in the VSLA-N@C and control groups, respectively.

**Table 1:** Characteristics of mothers, infants and their households

Characteristics	VSLA-N@C group (*N* = 53)	Control group (*N* = 50)	*P* -value
**Characteristics of households**			
*Food security*			
Food insecurity, *n* (%)	51 (96.2)	49 (98.0)	0.593
Food security, *n* (%)	2 (3.8)	1 (2.0)	
*Socio-economic status of households*			
High, *n* (%)	3 (5.8)	7 (14.0)	0.162
Poor, *n* (%)	49 (94.2)	43 (86.0)	
*Mean size of the households (SD)*	5.2 (1.4)	5.2 (1.7)	0.880
**Characteristics of mothers**			
Mean age of mothers, y (SD)	27.2 (5.6)	27.5 (6.9)	0.834
Mean BMI, kg/m^2^ (SD)	17.3 (1.7)	16.7 (1.7)	0.029
Mothers having at least a primary-level education, *n* (%)	23 (43.40)	20 (40.0)	0.727
Average number of children by mother (SD)	3.1 (1.7)	3.1 (1.5)	0.967
Member of VSLA, *n* (%)	53 (100)	31 (62.0)	0.000
Mothers living in a polygamous household, *n* (%)	14 (26.9)	20 (40)	0.199
**Characteristics of child**			
*Sex*			
Girls, *n* (%)	26 (49.1)	26 (52.0)	0.765
Boys, *n* (%)	27 (50.9)	24 (48.0)	
Mean age in months (SD)	4.57 (0.67)	5.05 (0.35)	0.000
Mean weight in kg (SD)	6.8 (1.0)	6.7 (1.0)	0.564
Mean length in cm (SD)	63.0 (2.7)	63.6 (2.6)	0.234
Mean LAZ (SD)	−0.43 (1.19)	−0.58 (1.1)	0.514
Mean WAZ (SD)	−0.16 (1.11)	− 0.61 (1.2)	0.055
Mean WLZ (SD)	0.23 (1.14)	−0.23 (1.0)	0.017

*VSLA* Village Saving and Loan Association

Among the mothers’ characteristics, there was a significant difference in mean BMI (*P* = 0.029) between the VSLA-N@C (17.3 ± 1.7 kg/m^2^) and the control group (16.7 ± 1.7 kg/m^2^). In the control group, 62.0% of mothers were members of VSLAs (but without N@C activities). The mean ages of the mothers were 27.2 ± 5.6 years and 27.5 ± 6.9 years in the VSLA-N@C and control groups, respectively.

Children’s age and mean WLZ differed significantly between zones. The mean age of the children was 4.57 ± 0.67 months in the VSLA-N@C group and 5.05 ± 0.35 months in the control group; the mean WLZ was a z-score of 0.23 ± 1.12 and a z-score of − 0.23 ± 1.0 in the VSLA-N@C and control groups, respectively.

### Evaluation of mothers’ participation in EBF promotion in the last 6 months

Table 2 shows mothers’ participation in EBF promotion activities. Apart from listening to radio messages on EBF promotion, in the VSLA-N@C group, the percentages of mothers who participated in activities promoted by the N@C project were greater than in the control group. The mothers in the intervention group more often followed those activities than mothers in the control group did (*P* = 0.000). This confirms that the intervention group has benefited from the intensive promotion of EBF.

**Table 2:** Mothers’ participation in activities promoting EBF during last 6 months

EBF activities promoted by the N@C project	VSLA-N@C group (*n* = 53)	Control group (*n* = 50)	*P*-value
1-Mothers listening to radio messages promoting breastfeeding, *n* (%)	28 (52.8)	31 (62.0)	0.347
Frequency of listening to the radio among mothers			
Seldom (one time)	8 (28.6)	15 (48.4)	0.002
Sometimes (two times)	7 (25.0)	14 (45.2)	
Often (three or more times)	13 (46.4)	2 (6.4)	
2-Mothers’ participation in BSCC sessions in the VSLA group on EBF, *n* (%)	53 (100)	16 (32.00)	0.000
Frequency of participation			
Seldom (one time)	3 (5.7)	13 (81.3)	0.000
Sometimes (two times)	13 (24.5)	3 (18.7)	
Often (three or more times)	37 (69.8)	0 (0.0)	
3-Mothers’ participation in BSCC sessions in the VSLA group on the best breastfeeding positions for optimal sucking, *n* (%)	49 (92.4)	12 (24.0)	0.000
Frequency of participation			
Seldom (one time)	4 (8.2)	8 (66.7)	0.000
Sometimes (two times)	14 (28.6)	3 (25.0)	
Often (three or more times)	31 (63.3)	1 (8.3)	
4-Mothers have received a visit from a community facilitator, *n* (%)	38 (71.7)	8 (16.0)	0.000
Seldom (one time)	6 (15.8)	5 (62.5)	0.010
Sometimes (two times)	18 (47.4)	3 (37.5)	
Often (three or more times)	14 (36.8)	0 (0.0)	
5-Individuals who have visited the mothers, *n* (%):			
N@C facilitator	36 (94.7)	0 (0.0)	0.000
Non-N@C facilitator	2 (5.3)	8 (100.0)	

*EBF* Exclusive breastfeeding, *VSLA* Village Loan and Saving Association, *BSCC* Behaviours and Social and Comportment Communication, *N@C* Nutrition at the Centre

### Human milk intake (HMI) and non-milk oral intake (NMOI) among infants by zone

Table 3 depicts the HMI and NMOI of the infants by zone. The mean infant HMI of the intervention group (900.2 ± 152.5 g/day) was significantly greater (*P* = 0.0443) than that of the control group (842.2 ± 188.6 g/day). The infants in the intervention group had lower non-milk oral intake than did those in the control group (*P* = 0.000). The difference in NMOI was 148.2 g/day.

**Table 3:** Human milk intake and non-milk oral intake by group

	VSLA-N@C group (*n* = 53)	Control group (*n* = 50)	Difference	*P* - value
Mean human milk intake ±SD, g/day	900.2 ± 152.5	842.2 ± 188.6	+ 58.0	0.044*
Median non-milk oral intake (25ie-75ie), g/day	95.0 (71.4–156.3)	243.5 (144.7–332.0)	−148.2	0.000**

*Two-sample *t*-test/**Median test, Pearson chi-square

### Exclusive breastfeeding practices by mothers

Table 4 shows the EBF practices by group and by method. The EBF rate measured by 24-h recall was greater than that measured by DTM in all groups (*P* = 0.000). The biases (differences in EBF rate between the two methods) were 58.5 and 64.0% in the intervention and control groups, respectively. No significant difference was found among these biases (*P* = 0.327). Therefore, the bias related to the methods did not depend on group. For all methods, the EBF rate was significantly higher in the intervention group than in the control group (*P* < 0.0001). By the DTM method, the EBF rates were approximately 38 and 8% in the intervention and control groups, respectively.

**Table 4:** EBF practices by mothers with 24-h recall and the deuterium oxide dose technique

Method of assessment mothers who practice EBF	VSLA-N@C group (*n* = 53)	Control group (*n* = 50)	Difference between groups	Significance between groups (*P*)
24-h recall, *n* (%)	51 (96.2) ^a^	36 (72.0) ^c^	+ 15 (+ 24.2)	0.001*
Deuterium dose-to-mother, *n* (%)	20 (37.7) ^b^	4 (8.0) ^d^	+ 16 (+ 29.7)	0.000*
Difference between methods, *n* (%)	31 (58.5)	32 (64.0)	−1 (−5.5)	0.327*

Values with different superscript letters in the same column are significantly different (*p* < 0.05); *chi-square test, comparison of two proportions; *EBF* Exclusive breastfeeding

### Association between EBF practices and VSLA-N@C project

Table 5 presents the association between zone and EBF rate, with adjustments made for membership in a VSLA, mothers’ BMI and children’s age, WAZ and WLZ. The multivariate logistic regression analysis revealed that the zone was significantly associated (*P* = 0.000) with the effective practice of EBF as measured by DTM. The mothers in the intervention group likely had effectively practised EBF almost 14 times more than those in the control group (adjusted OR = 13.9, 95% CI 1.6–116.5, *P* = 0.015). We obtained more evidence to find an association between the promotion of EBF and its practice with the DTM method than with the 24-h recall method (*P* = 0.015 vs *P* = 0.044).

**Table 5:** Association between EBF practices and participation in the VSLA N@C project

Parameter	OR Adjusted^a^	Z	*P* - value	95% CI
*Assessment of exclusive breastfeeding by deuterium oxide technique*^*1*^				
Group VSLA-N@C	13.9	2.42	0.015	1.6–116.5
*Assessment of exclusive breastfeeding by 24 h-recall*^*2*^				
Group VSLA-N@C	8.2	2.01	0.044	1.0–64.1

^a^Odd ratio adjusted by being a member of the VSLA, mothers’ BMI and children’s age, WAZ and WLZ; ^1^specification model: number of observations = 102; LR chi-square (6) = 20.03; log likelihood = −44.431591; pseudo R^2^ = 0.1839; ^2^specification model: number of observations = 102; LR chi-square (6) = 16.49; log likelihood = −36.06656; pseudo R^2^ = 0.1861

## Discussion

The main objective of this study is to verify, using the deuterium oxide dose-to-mother technique, whether mothers’ participation in the N@C project would improve the effectiveness of their EBF practices; the secondary objective was to document the process of N@C implementation. We found that the mothers who were members of VSLA-N@C effectively practised more EBF than those in the control group did (38% vs 8%). Considering the similarity in food security and socio-economic situation in the two zones, the observed difference could be explained by the difference in exposure to EBF promotion activities between the groups. Mothers in the intervention group have benefited significantly more from N@C activities, as outlined above (through home visits, behavioural and social change communication sessions and the large mobilization of the communities about issues related to breastfeeding). These activities that promote social behavioural change communication and effective EBF practices have significantly resulted in the high adherence of mothers in VSLAs to their recommended practices. Our results confirmed those of previous studies that showed that regular contact with advising agents and close monitoring were associated with a high rate of EBF practices. For example, in developing countries, family discussion sessions, home visits and video sessions facilitated the practice of EBF by mothers [28, 29].

On the other hand, we found that the discrepancy in EBF practices between the VSLA-N@C group and the control group, as determined by DTM, was greater than that revealed by the 24-h recall method. This highlights that we can track the effectiveness of the interventions more effectively if we use a precise method, such as stable isotope techniques. Further studies aiming to prove the efficacy of the interventions must use these reference methods, as mentioned in the publication of Owino [12].

One strength of our study is the age range of the children (4–5.5 months). It is generally known that the EBF rate is very low in this age group [23]. Beginning at 3 months of age, mothers usually regard infants as old enough to be left to other caregivers, particularly older siblings. The EBF rates found here (38% in VSLA-N@C by DTM) are very high compared to data from Benin’s last Demography Health Survey [5], which revealed a rate of 19.6% at 4–5 months of age. This value obtained in the DHS is close to 8%, as revealed by DTM in the control group, since the noticeable gap may be due to the difference in methods (DTM vs 24-h recall). Additionally, the large difference between the 19.6% (national level) and 38% (intervention group) values reinforces that N@C might affect EBF practices in this group.

Our study is a complement to the N@C endline evaluation [30] to reinforce the evidence that this integrated approach can help to improve EBF practices. In fact, the results from the endline evaluation showed that the EBF rate improved in the intervention group compared to the control group. From 2014 to 2018 in the intervention group, the EBF rate increased from 35.3 to 65.9%, and in the control group, the EBF rate decreased from 43.0 to 30.3% [30]. The explanation of these findings could be that the overall discussions about EBF practices with influential people (fathers, grandmothers and grandfathers) in households on the basis of teaching and a life trajectory following a monthly plan would contribute to decreased non-milk oral intake. These exchange spaces convey the message of breaking the sociological barriers that prevent the early introduction of water and/or other food and to provoke new behaviours within intervention groups.

Through our findings, children in the intervention group consumed more breast milk than those in the control group and mainly consumed less non-milk oral water. This could be explained by the fact that the mothers’ large degree of adherence to effective EBF practices was accompanied by an increase in the frequency and duration of breastfeeding. This information was part of the key messages broadcasted by the N@C during its communication sessions (“breastfeeding as needed” and “breastfeed the child until satiety”).

Moreover, even if human milk intake were significantly different between the two groups, these levels would not be clinically different, since 900 g vs 842 g per day are both values that are within normal ranges for exclusive breastfeeding at that age [31]. Another finding of our study and of the N@C intervention is not the difference in the quantities of breast milk intake but rather in the quantities of non-milk oral intake. Indeed, da Costa et al. [32] demonstrated that the introduction of 100 ml/day of water was associated with a decrease of 45 g/day in breast milk consumption by the child. In this study, we observed a gap of 148.2 g/day of water for a difference of 58 g/day in breast milk consumption between the children in each group. Thus, the increase in the proportion of mothers who exclusively breastfed was followed by an important decrease in non-milk oral intake in the intervention group compared to the control group.

The decrease in non-milk oral intake would drive potential improvement in paediatric health outcomes, such as the reduction of infectious diseases, among the children in the intervention group. We observed in our study that the children in the intervention group had a better nutritional status than the children in the control group (weight-for-age z-score: 0.23 vs − 0.23). This suggests a reduction in the risk of common childhood diseases (such as diarrhoea) in the intervention group. The introduction of even small amounts of complementary foods between zero and 6 months can be accompanied by certain hazards because it increases the risk of paediatric infectious disease and sudden infant death syndrome [21, 33, 34]. Further investigations are needed to focus on the effect of the N@C holistic approach (Fig. 1) or effective EBF practices on children’s body composition and nutritional status.

### Limitations of the study

One limitation of the study is the difference in the children’s ages (Table 1). The standard deviation for age in months is greater in the intervention group, suggesting that there were some children who were much younger in the intervention group than in the control group. Consequently, it is not surprising that the intervention group consumed slightly more breastmilk and fewer complementary foods. This limitation was considered during the analysis, and the logistic regression that was performed included age as a control variable.

The cut off value for non-milk oral intake (86.6 g/day) is much higher than the empirical value of 25 g/day used in most previous studies [6, 9–11, 14, 20, 21], using the DTM. The high value has been determined from a new model based on a distribution only of EBF children. This new model is still in development and it is not yet applicable to determine children who are not exclusively breastfed. The objective of our study is to determine the proportion of EBF children in each group at population level in order to compare them. In this case, this high value (86.6 g/day) would not be a problem.

Finally, as precise as the isotopic method may be, it only measures EBF rates over a 15-day period. It is possible that the mothers currently practised EBF only during the study. However, the children’s age of 4–5.5 months enables us to assert that our results are reliable. In fact, a child who usually consumes anything other than breast milk will not be able to go 15 days without consuming it. Thus, the mothers who exclusively breastfeed their children at 4–5.5 months of age, as revealed by the isotopic method, probably did so well before their children reached that age. The bias could be more present in breast milk consumption because mothers might possibly increase the frequency of breastfeeding. However, measuring EBF practices at different times during the 6 months as recommended would reinforce the conclusions of cross-sectional studies, which will never replace the gold-standard study.

## Conclusion

The mothers who participated in the N@C project through VSLA groups demonstrated an adherence to EBF practices that was almost fourteen times greater than that of mothers who did not. Additionally, the children of these mothers consumed more human milk and less water than did the children of the mothers in the control group. The N@C model could be promoted as a strategy for increasing EBF practices in poor and rural contexts, where it is possible to organize mothers into VSLA groups to discuss the process, benefits and challenges of exclusive breastfeeding.

## Data Availability

The datasets analysed during the current study are available from the corresponding author on reasonable request.

## Abbreviations

N@C: Nutrition at the Centre
EBF: Exclusive breastfeeding
DTM: Deuterium dose-to-mother
VSLA: Village saving and loan association
NMOI: Non-milk oral intake
HMI: Human milk intake
